# Clinical outcome and expression of mutant *P53*, *P16,* and *Smad4* in lung adenocarcinoma: a prospective study

**DOI:** 10.1186/s12957-015-0502-0

**Published:** 2015-03-28

**Authors:** Chunan Bian, Zhongyou Li, Youtao Xu, Jie Wang, Lin Xu, Hongbing Shen

**Affiliations:** Department of Thoracic Surgery, Nanjing Medical University-Affiliated Cancer Hospital, Nanjing, 210009 China; Jiangsu Key Laboratory of Molecular and Translational Cancer Research, Nanjing, 210009 China; Nanjing Jiangbei Peoples’ Hospital, Nanjing, 210048 China; The Public Health College of Nanjing Medical University, Nanjing, 210029 China

**Keywords:** Lung adenocarcinoma, Mutant *P53*, *P16*, *Smad4*, Immunohistochemistry, Prognosis

## Abstract

**Background:**

Whole-exome sequencing has shown that lung adenocarcinoma (LAC) can be driven by mutant genes, including *TP53*, *P16,* and *Smad4.* The aim of this study was to clarify protein alterations of *P53*, *P16,* and *Smad4* and to explore their correlations between the protein alterations and clinical outcome.

**Methods:**

We investigated associations among P53 mutant (P53^Mut^) expression, and P16 and Smad4 loss-of-expression, with clinical outcome in 120 LAC patients who underwent curative resection, using immunohistochemical (IHC) methods.

**Results:**

Of the 120 patients, 76 (63.3%) expressed P53^Mut^ protein, whereas 54 (45.0%) loss of P16 expressed and 75 (62.5%) loss of Smad4 expressed. P53^Mut^ expression was associated with tumor size (*P* = 0.041) and pathological stage (*P* = 0.025). Loss of P16 expression was associated with lymph node metastasis (*P* = 0.001) and pathological stage (*P* < 0.001). Loss of Smad4 expression was associated with tumor size (*P* = 0.033), lymph node metastasis (*P* = 0.014), pathological stage (*P* = 0.017), and tumor differentiation (*P* = 0.022). Kaplan-Meier survival analysis showed that tumor size (*P* = 0.031), lymph node metastasis (*P* < 0.001), pathological stage (*P* < 0.001), P53^Mut^ protein expression (*P* = 0.038), and loss of p16 or Smad4 expression (*P* < 0.001) were significantly associated with shorter overall survival(OS), whereas multivariate analysis indicated that lymph node metastasis (*P* = 0.014) and loss of p16 or Smad4 expression (*P* < 0.001) were independent prognostic factors. Analysis of protein combinations showed patients with more alterations had poorer survival (*P* < 0.001). Spearman correlation analysis showed that loss of Smad4 expression inversely correlated with expression of P53^Mut^ (*r* = ^−^0.196, *P* = 0.032) and positively with lost P16 expression (*r* =0.182, *P* = 0.047).

**Conclusions:**

The findings indicate that IHC status of P53^Mut^, P16, and Smad4 may predict patient outcomes in LAC.

## Background

Lung cancer is the leading cause of cancer death worldwide [1], and the proportion of lung cancer patients with lung adenocarcinoma (LAC) is reportedly increasing [2]. Survival rates for LAC have improved dramatically in the last decade, owing to identification of driver mutations in LAC [3]. For example, EGFR tyrosine kinase inhibitor have been approved for treatment of LACs that carry *EGFR* gene mutations, which has greatly improved the prognosis of such patients [4,5]. Discovery of genetic biomarkers for cancers is expected to rapidly expand [6]. Identified driver gene alteration for LAC currently includes mutations of *EGFR*, *KRAS*, *PIK3CA*, *BRAF*, *STK11*, *DDR2*, *TP53*, *Smad4*, *P16*, *RET*, and *ALK*, among others [7-11]. As driver genes are found, their relationships to each other and to patients’ prognoses must be verified.

Genetic alterations of *P53*, *P16*, and *Smad4* have been found in pancreatic cancer, and appear to be strongly associated with its malignant behavior [12-14]. In our previous study with a genetically engineered mouse model, we found P53^Mut^’s potentially malignant gain-of-function was promoted by inactivating the inhibitory actions of transforming growth factor β (TGF-β), caused by downregulation of smad4, which in turn was synergistically caused by *P53*^Mut^ and deficient *P16*/*P19*. Although these three genes have been studied individually in LAC, little is known about how they interact, or their combined effect on prognosis. Here, we investigated mutant *P53*, *P16*, and *Smad4* in LAC by immunohistochemical (IHC) staining and correlated these mutations with clinicopathological features and patients’ OS.

## Methods

### Patients and tissue samples

This study included 120 patients with LAC who underwent surgical resection between January 2007 and March 2009 at the Nanjing Medical University-Affiliated Cancer Hospital, Nanjing, China. All patients had complete medical records and complete follow-up data. The last follow-up date was March 2014. Patients who died of causes other than LAC before this date were excluded. Their clinicopathological data were collected from medical records and follow-up data were obtained through telephone interviews or by consulting the police population information system. These patients’ mean age was 59.4 years (range: 35 to 85 years), including 58 men and 62 women. Before their surgeries, all patients underwent CT scans or B-ultrasonic examinations to exclude locoregional or widespread metastases. All patients underwent radical resections; no patients received radiotherapy or chemotherapy before surgery. This study was approved by the Ethics Committee of Nanjing Medical University.

### IHC analyses

**Figure 1:**
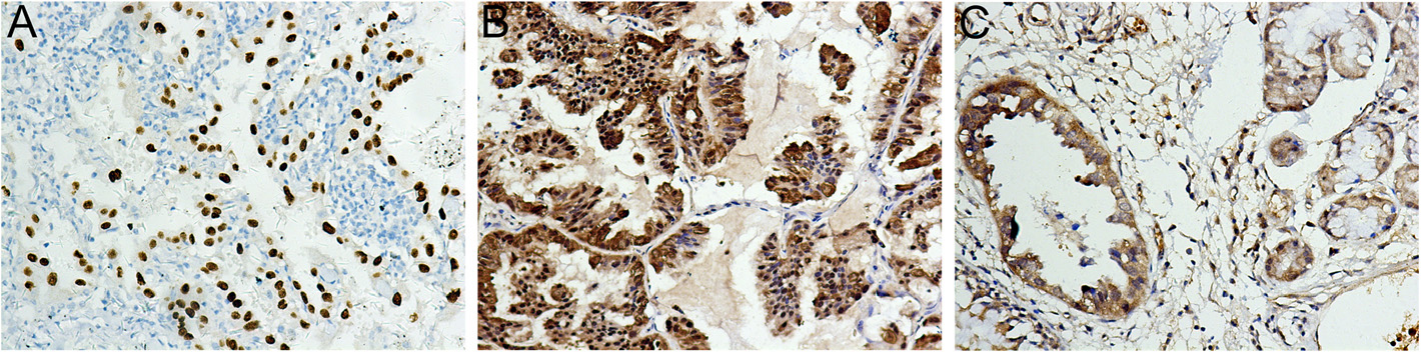
**P53** ^**Mut**^ **, P16, and Smad4 expression in lung adenocarcinoma, shown immunohistochemically (SP × 200). (A)** P53^Mut^ positive staining detected in nucleus. **(B)** P16 positive staining detected in cytoplasm and nucleus. **(C)** Smad4 positive staining detected in cytoplasm and nucleus.

Specimens of primary LAC from 120 patients were cut into 5-μm tissue sections and deparaffinized by routine methods. The slides were steamed for 20 min in sodium citrate buffer. After cooling for 5 min, the slides were IHC stained for P53^Mut^, P16 and Smad4. At least five different distinct regions of the primary tumor were IHC-labeled for each case to evaluate for potential heterogeneity. IHC labeling was carried out using *P53*^Mut^ mouse monoclonal antibody (clone SC126 diluted 1:100, Santa Cruz Biotechnology, Dallas, USA), CDKN2A/P16 rabbit monoclonal antibody (clone SC468 diluted 1:100, Santa Cruz Biotechnology), and Smad4/Dpc4 mouse monoclonal antibody (clone SC-7966, diluted 1:100, Santa Cruz Biotechnology) as reported [15]. Labeling was detected by adding biotinylated secondary antibodies. Positive controls were taken from sections known to be positive from pancreatic carcinoma specimens. For the negative controls, 1% PBS was used in place of primary antibodies. Results were evaluated independently by two experienced pathologists. P53^Mut^ was considered positive when ≥10% of tumor cell nuclei showed strong staining with a dark brown color. P16 and Smad4 were considered positive when ≥ 20% of tumor cell cytoplasm and nuclei showed staining with a brown color (Figure 1).

### Statistical analysis

Statistical analysis of group differences was performed using *χ*^2^ tests. The 1-, 3-, and 5-year survival rates were estimated using life tables; OS was estimated using the Kaplan-Meier method, and the differences were assessed by the log-rank test. Cox proportional hazards models were generated for multivariate analysis. Correlation analysis used the Spearman test. *P* < 0.05 was considered statistically significant. Statistical analyses were performed using SPSS software (version 17.0, SPSS).

## Results

### Clinicopathological features and outcome

Of the 120 patients (58 men and 62 women), 47 (39.2%) were older than 60 years at the time of surgery; their mean and median ages were 59.4 and 58 years, respectively. At the last follow-up date (March 2014), 25 (20.8%) patients were still alive. Median OS was 35.14 months, with 1-, 3-, and 5-year survival rates of 61.0%, 39.0%, and 33.0%, respectively. In all 120 patients, 24 (20.0%) had T1 tumors, 73 (60.8%) had T2 tumors, and 23 (19.2%) had T3/4 tumors. Lymph node metastases were present in 49/120 (40.8%). We found 26.7% of tumors were well differentiated, 34.1% were moderately differentiated, and 39.2% were poorly differentiated. Only 13 (10.8%) patients had pleural invasion. Of the 120 patients, 37 (30.8%), 47 (39.2%), 36 (30.0%), and 0 (0%) presented with the Union for International Cancer Control stage I, II, III and IV disease, respectively (Table 1).

**Table 1:** **Mutant** ***P53*** **,** ***P16*** **, and** ***Smad4*** **expression in relation to clinicopathological parameters (** ***n*** **= 120)**

	**Mutant** ***P53*** **expression**		***P***	***P16*** **expression**		***P***	***Smad4*** **expression**		***P***
	**Negative (%)**	**Positive (%)**		**Negative (%)**	**Positive (%)**		**Negative (%)**	**Positive (%)**	
Age ≤60 year	26 (35.6%)	47 (64.4%)	0.766	34 (46.6%)	39 (53.4%)	0.666	47 (64.4%)	26 (35.6%)	0.595
Age >60 year	18 (38.3%)	29 (61.7%)		20 (42.6%)	27 (57.4%)		28 (59.6%)	19 (40.4%)	
Male	25 (43.1%)	33 (56.9%)	0.157	27 (46.6%)	31 (53.4%)	0.741	32 (55.2%)	26 (44.8%)	0.109
Female	19 (30.6%)	43 (69.4%)		27 (43.5%)	35 (56.5%)		43 (69.4%)	19 (30.6%)	
Tumor size (cm)									
T1 (≤3)	14 (58.3%)	10 (41.7%)	*0.041*	8 (33.3%)	16 (66.7%)	0.392	11 (45.8%)	13 (54.2%)	*0.033*
T2 (>3 ≤ 7)	24 (32.9%)	49 (67.1%)		34 (46.6%)	39 (53.4%)		45 (61.6%)	28 (38.4%)	
T3/4 (>7)	6 (26.1%)	17 (73.9%)		12 (52.2%)	11 (47.8%)		19 (82.6%)	4 (17.4%)	
Lymph nodes									
Negative	27 (38.0%)	44 (62.0%)	0.709	23 (32.4%)	48 (67.6%)	*0.001*	38 (53.5%)	33 (46.5%)	*0.014*
Positive	17 (34.7%)	32 (65.3%)		31 (63.3%)	18 (36.7%)		37 (75.5%)	12 (24.5%)	
Differentiation									
Well	16 (50.0%)	16 (50.0%)	0.188	13 (40.6%)	19 (59.4%)	0.077	15 (46.9%)	17 (53.1%)	*0.022*
Moderate	13 (31.7%)	28 (68.3%)		14 (34.1%)	27 (65.9%)		24 (58.5%)	17 (41.5%)	
Poor	15 (31.9%)	32 (68.1%)		27 (57.4%)	20 (42.6%)		36 (76.6%)	11 (23.4%)	
Pleural invasion									
Negative	39 (36.4%)	68 (63.6%)	0.887	47 (43.9%)	60 (56.1%)	0.497	66 (61.7%)	41 (38.3%)	0.596
Positive	5 (38.5%)	8 (61.5%)		7 (53.8%)	6 (46.2%)		9 (69.2%)	4 (30.8%)	
Pathological stage									
Stage I	20 (54.1%)	17 (45.9%)	*0.025*	11 (29.7%)	26 (70.3%)	*<0.001*	18 (48.6%)	19 (51.4%)	*0.017*
Stage II	15 (31.9%)	32 (68.1%)		17 (36.2%)	30 (63.8%)		28 (59.6%)	19 (40.4%)	
Stage III	9 (25.0%)	27 (75.0%)		26 (72.2%)	10 (27.8%)		29 (80.6%)	7 (19.4%)	

The italicized values indicate *P* values less than 0.05.

### Protein alterations in LAC

Using IHC labeling, we detected positive P53^Mut^ in 76 patients (63.3%), negative P16 in 54 patients (45.0%), negative Smad4 in 75 patients (62.5%) (Table 1), alterations of all three proteins (P53mut+/P16-/Smad4-) in 28 (23.3%) patients, and normal expression of the three proteins (P53mut-/P16+/Smad4+) in 17 (14.2%) patients (Table 2).

**Table 2:** **Clinicopathological parameters and overall survival in 120 patients**

		**Mean (month)**		**Median (month)**		
	***N***	**Estimate**	**95**% **CI**	**Estimate**	**95%** **CI**	***P***
Age (year)						
≤60	73	41.520	35.148 to 47.892	37.000	24.907 to 49.093	0.883
>60	47	40.396	32.890 to 47.902	30.000	17.931 to 42.069	
Sex						
M	58	41.563	34.672 to 48.454	36.000	18.585 to 53.415	0.917
F	62	40.535	33.685 to 47.385	30.000	17.461 to 42.539	
Tumor size (cm)						
T1 (≤3)	24	49.125	40.950 to 57.300	45.000	31.797 to 58.203	*0.031*
T2 (3 to 7)	73	41.959	35.673 to 48.245	35.000	17.325 to 52.675	
T3/4 (>7)	23	28.587	17.986 to 39.188	15.000	10.340 to 19.660	
Lymph nodes						
Negative	71	50.437	44.488 to 56.387	49.000	39.870 to 58.130	*<0.001*
Positive	49	27.497	20.990 to 34.003	17.000	13.571 to 20.429	
Differentiation						
Well	32	48.869	40.548 to 57.190	49.000	33.785 to 64.215	0.067
Moderate	41	43.975	35.538 to 52.412	35.000	16.180 to 53.820	
Poor	47	33.009	25.461 to 40.556	21.000	14.283 to 27.717	
Pleural invasion						
Negative	107	42.446	37.213 to 47.680	37.000	25.020 to 48.980	0.091
Positive	13	31.385	18.335 to 44.434	23.000	15.954 to 30.046	
Stage						
I	37	57.224	49.411 to 65.036	55.000	42.364 to 67.636	*<0.001*
II	47	39.400	31.965 to 46.836	30.000	20.613 to 39.387	
III	36	27.111	19.609 to 34.613	16.000	11.296 to 20.704	
Mutant *P53*						
Negative	44	47.794	39.707 to 55.881	47.000	29.665 to 64.335	*0.038*
Positive	76	37.172	31.317 to 43.027	30.000	20.511 to 39.487	
*P16*						
Negative	54	28.352	22.689 to 34.014	20.000	14.399 to 25.601	*<0.001*
Positive	66	51.359	44.929 to 57.788	56.000	40.849 to 71.151	
*Smad4*						
Negative	75	30.185	25.074 to 35.297	21.000	15.346 to 26.654	*<0.001*
Positive	45	59.678	52.491 to 66.866	66.000	50.912 to 81.088	
Gene expression combinations						*<0.001*
*P53mut−/P16+/Smad4−*	11	43.364	27.766 to 58.961	47.000	13.553 to 80.447	
*P53mut−/P16+/Smad4+*	17	63.824	54.411 to 73.236	74.000	44.191 to 103.809	
*P53mut−/P16−/Smad4+*	5	53.800	33.542 to 74.058	49.000	38.265 to 59.735	
*P53mut−/P16−/Smad4−*	11	22.000	14.791 to 29.209	21.000	14.746 to 27.254	
*P53mut+/P16+/Smad4−*	25	41.451	31.906 to 50.997	32.000	12.416 to 51.584	
*P53mut+/P16+/Smad4+*	13	58.011	44.148 to 71.874	66.000	38.979 to 93.021	
*P53mut+/P16−/Smad4+*	10	52.100	38.216 to 65.984	54.000	27.658 to 80.342	
*P53mut+/P16−/Smad4−*	28	18.185	14.057 to 22.313	15.000	12.976 to 17.024	

The italicized values indicate *P* values less than 0.05.

### Protein alterations and clinicopathological features

Positive IHC labeling of P53^Mut^ was significantly linked to tumor size (*P* = 0.041) and pathological stage (*P* = 0.025). Negative P16 IHC labeling was significantly associated with lymphatic metastasis (*P* = 0.001) and pathological stage (*P* < 0.001). Negative Smad4 IHC labeling was associated with tumor size (*P* = 0.033), lymph node metastasis (*P* = 0.014), differentiation (*P* = 0.022), and pathological stage (*P* = 0.017) (Table 1).

### Clinicopathological features and OS

Univariate analysis results were based on log-rank tests of clinicopathological characteristics in relation to OS. Tumor size (*P* = 0.031), lymph node metastasis (*P* < 0.001), and pathological stage (*P* < 0.001) were significantly associated with shorter OS (Table 2).

### Protein alterations and OS

**Figure 2:**
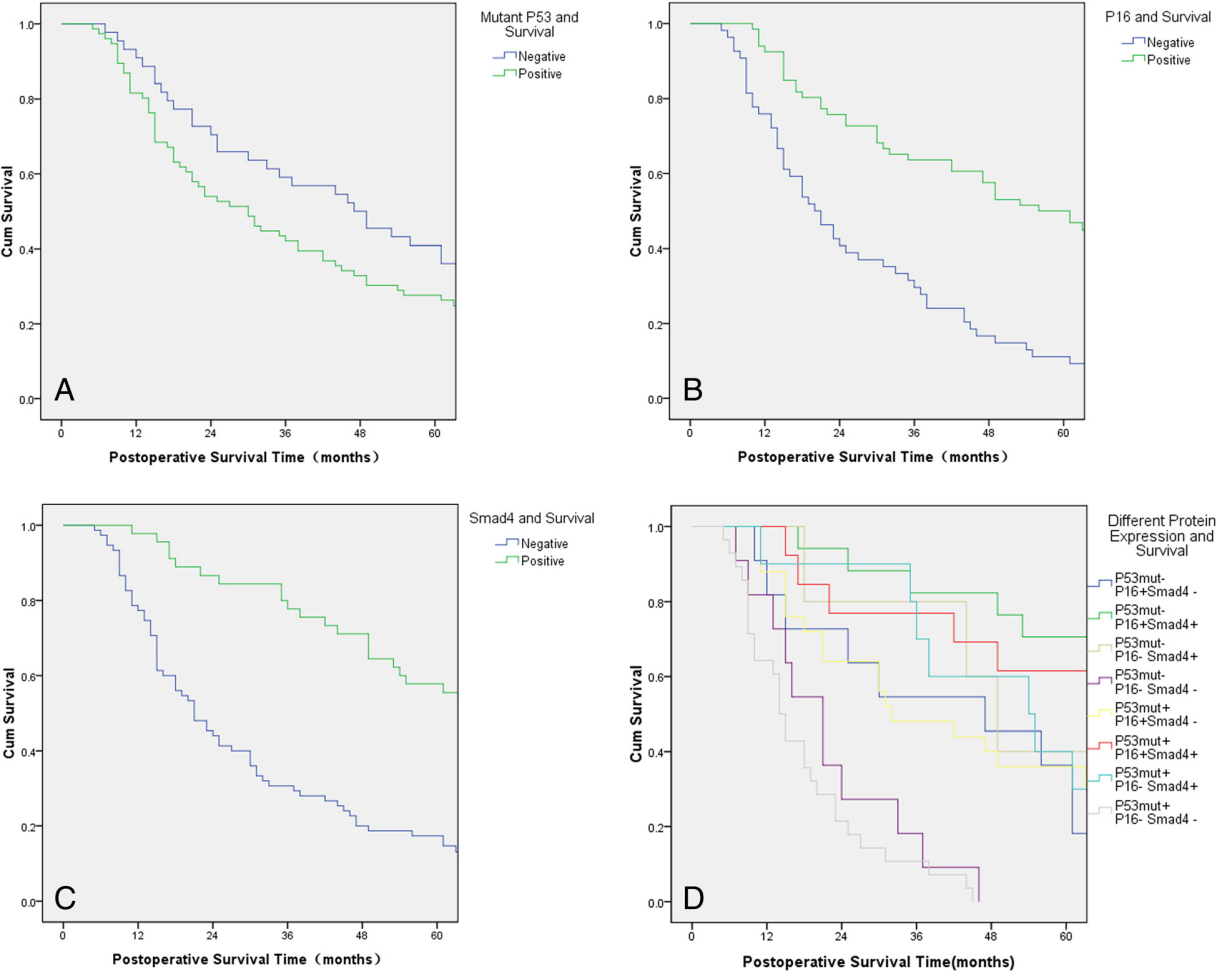
**Changes in protein expression and overall survival (OS). (A)** Patients’ postoperative OS curves by *P53* ^Mut^ expression. **(B)** Patients’ postoperative OS curves by *P16* expression. **(C)** Patients’ postoperative OS curves by *Smad4* expression. **(D)** Patients’ postoperative OS curves by different protein expression combinations.

Loss of P16 and Smad4 IHC labeling was associated with a significantly shorter OS (*P* < 0.001). There were significant differences in positive labeling of P53^Mut^ with regard to OS (*P* = 0.038). Next, based on the number of altered proteins, we classified the patients into eight groups: *P53mut*−/*P16*+/*Smad4*− (*n* = 11); *P53mut*−/*P16*+/*Smad4*+ (*n* = 17); *P53mut*−/*P16*−/*Smad4*+ (*n* = 5); *P53mut*−/*P16*−/*Smad4*− (*n* = 11); *P53mut*+/*P16*+/*Smad4*− (*n* = 25); *P53mut*+/*P16*+/*Smad4*+ (*n* = 13); *P53mut*+/*P16*−/*Smad4*+ (*n* = 10); and *P53mut*+/*P16*−/*Smad4*− (*n* = 28). Kaplan-Meier survival analysis showed that the *P53mut*−/*P16*+/*Smad4*+ group had the longest OS and the *P53 mut*+/*P16*−/*Smad4*- group had the shortest OS (*P* < 0.001). The higher number of altered proteins robustly reflected major differences in survival outcome. The results showed patients with more protein alterations had poorer survival rates (Table 2, Figure 2).

### Multivariate analyses of factors affecting OS

Multivariate models using Cox proportional hazards analysis were conducted with the parameters that were significant at the *P* < 0.05 level on univariate analysis using log-rank tests. Multivariate analysis showed that lymph node metastasis (relative risk (RR): 2.222, *P* = 0.014), negative Smad4 IHC labeling (RR: 0.269, *P* < 0.001) and negative P16 IHC labeling (RR: 0.360, *P* < 0.001) were independent predictors of OS (Table 3).

**Table 3:** **Multivariate analysis of prognostic factors for overall survival (** ***n*** **= 120)**

**Factors**	**Relative risk**	**95**% **CI**	***P***
Tumor size	1.423	0.990 to 2.044	0.056
Status of lymph nodes metastasis	2.222	1.172 to 4.210	*0.014*
Pathological stage	1.075	0.706 to 1.636	0.735
Positive *P53* ^*Mut*^ labeling	1.320	0.834 to 2.091	0.236
Negative *P16* labeling	0.360	0.226 to 0.572	*<0.001*
Negative *Smad4* labeling	0.269	0.165 to 0.441	*<0.001*

The italicized values indicate *P* values less than 0.05.

### Correlation analysis of P53^mut^, P16 and Smad4 expression

Spearman analysis indicated that Smad4 expression was negatively correlated with P53^mut^ expression (*r* = −0.196, *P* = 0.032) and positively correlated with P16 expression (*r* = 0.182, *P* = 0.047), whereas P16 expression and P53^mut^ expression showed no correlation (Table 4).

**Table 4:** **Relationships among mutant** ***P53*** **,** ***P16*** **, and** ***Smad4***

	***TP53***	***P16***	***Smad4***
Mutant *P53*			
*r*	1.000	−0.132	−0.196
*P*	-	0.150	*0.032*
*P16*			
*r*	−0.132	1.000	0.182
*P*	0.150	-	*0.047*
*Smad4*			
*r*	−0.196	0.182	1.000
*P*	*0.032*	*0.047*	-

The italicized values indicate *P* values less than 0.05; *r*, Pearson correlation.

## Discussion

The molecular basis of lung cancer is complex and heterogeneous. Over the last decades, identification of driver mutations in LAC has led to the development of targeted agents, several of which are in clinical trials and are already approved for clinical use [6].

Recent whole-exome sequencing studies of numerous human cancers have conclusively shown TP*53* to be the most frequently mutated gene in human cancers [15,16]. The P53 protein and its downstream pathways are important in preventing tumor formation, but TP*53* mutation is common in cancers. Moreover, unlike other tumor-suppressor genes that only lose their tumor-suppressor functions, the *P53*^Mut^ gene may endow its mutant protein with new activities that actively promote tumor progression and increased resistance to anticancer treatments [17]. Because P53^Mut^ proteins have longer half-life than wild-type P53, which can accumulate in the nucleus, we only can detect P53^Mut^ proteins by IHC. Although Ding *et al*. found that 45% of LAC patients had *TP53* mutations [18], the clinical implications of mutant P53 in LAC may still be conflicting. *P53* alterations are reported to predict poor survival in patients with non-small cell lung cancer (NSCLC) [19-22]. However, Ahn *et al*. reported that P53^Mut^ protein expression did not correlate with OS in NSCLC [23]. In our study, the *P53*^Mut^ frequency was 63.3%, which was higher than that previously reported. The results of univariate analyses showed that higher P53^Mut^ IHC expression predicted shorter OS. However, the multivariate analysis indicated that higher P53^Mut^ expression did not independently predict poorer OS. Furthermore, mice that express P53^Mut^ reportedly have a more aggressive and metastatic tumor profile than that of mice with null or wild-type *P53* [24,25]. Conversely, Jackson *et al*. reported that *P53*^Mut^ protein in lung showed no detectable gain-of-function activity [26]. Although the present study found no relationship between P53^Mut^ and lymph node metastasis, P53^Mut^ expression was linked to tumor size and pathological stage. The role of TP53 mutations as a prognostic marker in NSCLC were reported conflicting. This may be due to the molecular heterogeneity and differing functional effects specific to various TP53 genotypes, methodological issues related to the assessment of mutation status, and design issues related to small sample size and nonhomogeneous groups of patients. Furthermore, the context in which these mutations occur, the initiating events and other secondary molecular alterations, may matter as well. Hence, a larger sample size and more complete experiment methods will be required in the future to obtain reliable and consistent results.

*P16* is an important tumor-suppressor gene that has been found to affect cell-cycle by inactivating the cyclin-dependent kinase inhibitor [27-29]. *P16* alterations in NSCLC were mainly homozygous deletions, promoter hypermethylation and point mutations [30]. The relationship between P16 expression and lung cancer is still unclear. Although some studies reported that P16 expression increased in NSCLC [31,32], another found the *P16* gene to be a commonly inactivated tumor-suppressor gene in NSCLC, and altered *P16* and *P53* genes to be frequently found in the same tumors [30]. In this study, loss of P16 was linked to lymph node metastasis and pathological stage, which accords with the study that found complete P16 inactivation in advanced NSCLC [30].

*Smad4* is a tumor-suppressor gene with a key role in the TGF-β signaling pathway [33]. Because of its mediatory role in the growth-inhibitory effects of TGF-β in normal cells and its loss in some tumors, *Smad4* is considered a tumor-suppressor gene [34]. Alterations of *Smad4* gene were reported in pancreatic, colorectal, gastric, esophageal, and breast tumors; its loss is associated with tumorigenesis and progression [35-39]. However, the role of Smad4 in LAC is unclear. NSCLC reportedly features low Smad4 expression, which is closely correlated with lymph node metastasis but not with histological type or differentiation [40]. Our study found the loss of *Smad4* was key to LAC occurrence and development; our IHC results showed a 62.5% loss rate for Smad4 in patients with LAC. Negative Smad4 labeling was associated with tumor size, lymph node metastasis, differentiation, and pathological stage, and patients with Smad4 negative specimens had worse OS. Thus, reduced Smad4 expression in LAC may predict poor prognosis.

We also found that patients with more protein alterations had worse OS. Possibly, accumulated protein alterations greatly influence LAC development; this would also indicate that combinations of protein alterations are more accurate predictors for patient outcome than single alterations.

We used Spearman correlation analysis to investigate the relationship among smad4, P53^Mut^, and P16. Although expressions of Smad4 and P53^Mut^ were inversely correlated, Smad4 expression was positively correlated with P16 expression. A previous study revealed that in SMMC-7221 hepatocellular carcinoma cells, TGF-β inhibited proliferation by upregulating P16 expression and increased apoptosis by activating caspase 3 in a Smad4-dependent manner [41]. Another study showed that low Smad4 expression is related to the high p53 expression in breast tumors [42]. As these results are similar to ours, we speculated that LAC could have a similar mechanism. In another study with results that accorded with ours, knocked-down *P53* (using siRNA) reportedly increased Smad4 activity and promoted apoptosis in MCF-7 breast cancer cells [39]. Montserrat *et al*. found that *P16* was a commonly inactivated tumor-suppressor gene in NSCLC and that *P16* alterations and *P53* mutations were frequently found in the same tumor [30]. However, in this paper, we found no correlation between P16 expression and TP53 expression.

## Conclusions

In conclusion, alterations of the *P53*, *P16,* and *Smad4* proteins were strongly associated with LAC malignancy of LAC. Their IHC assessment at the time of diagnosis may provide a new prognostic method, assisting in deciding optimal treatment strategies for patients with LAC.

## Abbreviations

IHC: immunohistochemical
LAC: lung adenocarcinoma
NSCLC: non-small cell lung cancer
OS: overall survival
TGF-β: transforming growth factor β
